# Statistical challenges in modelling the health consequences of social mobility: the need for diagonal reference models

**DOI:** 10.1007/s00038-017-1018-x

**Published:** 2017-07-17

**Authors:** Jeroen van der Waal, Stijn Daenekindt, Willem de Koster

**Affiliations:** 1 Department of Public Administration and Sociology, Erasmus University Rotterdam, Rotterdam, The Netherlands; 2 Department of Sociology, Ghent University, Ghent, Belgium

**Keywords:** Social mobility, Mobility effects, Socio-economic position (SEP), Status inconsistency, Diagonal reference models (DRMs), Obesity

## Abstract

**Objectives:**

Various studies on the health consequences of socio-economic position address social mobility. They aim to uncover whether health outcomes are affected by: (1) social mobility, besides, (2) social origin, and (3) social destination. Conventional methods do not, however, estimate these three effects separately, which may produce invalid conclusions. We highlight that diagonal reference models (DRMs) overcome this problem, which we illustrate by focusing on overweight/obesity (OWOB).

**Methods:**

Using conventional methods (logistic-regression analyses with dummy variables) and DRMs, we examine the effects of intergenerational educational mobility on OWOB (BMI ≥ 25 kg/m^2^) using survey data representative of the Dutch population aged 18–45 (1569 males, 1771 females).

**Results:**

Conventional methods suggest that mobility effects on OWOB are present. Analyses with DRMs, however, indicate that no such effects exist.

**Conclusions:**

Conventional analyses of the health consequences of social mobility may produce invalid results. We, therefore, recommend the use of DRMs. DRMs also validly estimate the health consequences of other types of social mobility (e.g. intra- and intergenerational occupational and income mobility) and status inconsistency (e.g. in educational or occupational attainment between partners).

## Introduction

The social gradient in health is well-established (Mackenbach et al. 2008). One aspect of socio-economic position (SEP) that is likely to affect health outcomes is ‘social mobility’, which denotes downwards or upwards movement on the social ladder from one’s ‘position of origin’ to one’s ‘position of destination’. This movement in positions can occur within (i.e. intragenerational mobility) or between (i.e. intergenerational mobility) generations, and can pertain to various aspects of SEP, e.g. occupational, educational and income mobility. These various forms of social mobility can affect individuals health in several ways.

Social mobility effects have been studied with respect to a wide range of health outcomes and behaviours (Boyle et al. 2009; Calvo and Morrison 2016; Cardano et al. 2004; Hart et al. 2008; Janicki-Deverts et al. 2011; Kawachi et al. 2008), with a predominant focus on negative effects on health. This focus is not unexpected as social mobility may be associated with, for instance, being deprived of social networks (Lundberg 1991). It may also lead to stress that arises from being uprooted from one’s social environment of origin and/or not fitting into a new social environment (Bourdieu 2000).

It should be noted that mobility effects refer to the consequences of experiencing social mobility itself, aside from the effects of one’s social positions of origin and destination. If socially mobile individuals, for instance, adopt patterns of diet and exercise that are characteristic of their newly acquired social position, the health effects do not originate from social mobility as such, but are instead due to how an individual adapts to their social position of destination. By demonstrating how social mobility effects can be studied, this article aims to contribute to the validity of future research on social mobility in public health. For this purpose, we take the effects of intergenerational social mobility on OWOB as an illustration.

At least 15 studies have previously estimated the effects of intergenerational social mobility on body mass index (BMI) or overweight/obesity (OWOB), 13 of which claim to find empirical support for their existence (Aitsi-Selmi et al. 2013; Ball and Mishra 2006; Barros et al. 2006; Blane et al. 1996; Boylan et al. 2014; Chaparro and Koupil 2014; Gigante et al. 2008; Goldblatt 1965; Heraclides and Brunner 2009; James et al. 2006; Kavikondala et al. 2009; Krzyzanowska and Mascie Taylor 2011; Kuntz and Lampert 2012; Langenberg et al. 2003; Muraro et al. 2016). These studies apply a conventional approach in public health research: differentiating groups based on combinations of their position of origin and position of destination, and subsequently comparing the BMI or OWOB scores between those groups. However, as discussed below, this approach does not allow to empirically disentangle mobility effects from origin and destination effects.

We highlight an alternative approach that has been underutilized in public health: so-called Diagonal Reference Models (DRMs; originally denoted as ‘Diagonal Mobility Models’; Sobel 1981, 1985). These models were especially developed for the purpose of empirically disentangling mobility effects from origin and destination effects. Of the abovementioned studies on the effects of intergenerational mobility on BMI or OWOB, only Chaparro and Koupil rightly pointed out the need for using this method when studying mobility effects. They were unable to apply it in their research themselves, because they studied social mobility across three generations, while DRMs do not allow more than two generations to be included in an analysis. Despite this limitation, DRMs hold great promise for public health research.

We aim to demonstrate the value of DRMs for estimating social mobility effects in public health studies, and we provide suggestions for their future application. The underutilization of DRMs in public health is probably related to the fact that this method is not included in standard statistical software packages, and that it is, therefore, absent from standard university curricula. Fortunately, researchers are able to use DRMs relatively easily, because relevant packages and scripts have recently been developed. These include Tolsma et al.’s *SPSS* tutorial (2009: 266), Turner and Firth’s *Dref* subcommand of the *gnm R* package (2007), and Lizardo’s *Stata* package (2007).

We illustrate the advantages of DRMs in social mobility research in public health by contrasting this method with conventional approaches applied in the field. For brevity’s sake our study will only focus on the OWOB effects of intergenerational educational mobility, i.e. the discrepancy between the educational level of adults and that of their parents. This is also the focus of several of the mobility studies on BMI or OWOB discussed above (e.g. Boylan et al. 2014; Kuntz and Lampert 2012). However, our methodological argument also applies to other types of social mobility and to mobility effects on health outcomes other than OWOB. This means that the implications of the problems addressed in this study are relevant for social mobility studies in public health in general.

## Methods

### Data

To illustrate our argument empirically, we used the first wave of the NEtherlands Longitudinal Lifecourse Study (NELLS), which is representative of the Dutch population aged 15–45 in 2009 (*n* = 5312) (De Graaf et al. 2009). Two-stage stratified sampling was applied: (1) 35 municipalities were selected, and (2) a random selection from the population registry was made based on age and the country of birth of the respondents and their parents. Those of Moroccan and Turkish origin were oversampled, and so we applied the weight factor provided by the data collector to adjust for this.

### Measures

In line with previous studies on the relationship between intergenerational educational mobility and OWOB (e.g. Kuntz and Lampert 2012), we measured parental education using the educational level of the parent who had achieved the highest qualification. Sensitivity analyses show that measuring it with only the educational level of the father (e.g. Boylan et al. 2014; Chaparro and Koupil 2014) leads to similar conclusions as the results reported below: regardless of the operationalisation of parental education, conventional analyses indicate various mobility effects, while DRMs demonstrate that no mobility effects are present. Based on the International Standard Classification of Education 2011 (ISCED), we classified parental educational level into three categories: (1) low (categories 1–2), (2) medium (categories 3–4), and (3) high (categories 6–8). The educational level of the respondents in our study was measured using the same categories. Individuals still enrolled in the education system were omitted from the analyses. Table 1 provides the mobility trajectories of all the respondents included in the analyses.

**Table 1:** Overview of intergenerational educational mobility among males and females, The Netherlands 2009

	Males				Females			
	Destination							
Origin	Low	Medium	High	Total	Low	Medium	High	Total
Low	**361**	341	142	844	**347**	414	126	887
Medium	68	**220**	137	425	68	**324**	172	564
High	17	112	**171**	300	18	103	**199**	320
Total	446	673	450	**1569**	433	841	497	**1771**

Immobile individuals on diagonals (bold), downwardly mobile individuals below diagonals, upwardly mobile individuals above diagonals

Using the International Standard Classification of Education 2011, educational level is classified into three categories: (i) low (categories 1–2), (ii) medium (categories 3–4), and (iii) high (categories 6–8)

Like the studies on mobility and BMI or OWOB discussed above, we analysed males and females separately. OWOB was defined as a BMI ≥ 25 kg/m^2^ (45.4% OWOB) and was calculated based on a respondent’s self-reported height and weight. Following the bulk of the studies discussed in the Introduction, we omitted individuals younger than 18 years from the analyses to measure OWOB validly. For the remaining respondents, age (in years) was included as a control variable and was centred around its mean of 34.95 (SD = 6.88). We also controlled for marital status (‘no partner’ 19.1%; ‘married and cohabiting’ 56.4%; ‘unmarried and cohabiting’ 17.0% and ‘not cohabiting, married or unmarried’ 7.5%). In addition, we included a control variable indicating whether the respondents were natives of the Netherlands (52.6%) or non-natives. Individuals were considered to be the latter if at least one of their parents was born outside the country.

### Contrasting conventional methods to Diagonal Reference Models

A conventional method in public health research for studying social mobility effects consists of differentiating groups based on combinations of their position of origin and position of destination. There are three educational levels in our study, which means that there are nine (3 × 3) mobility groups (see Table 1). In this conventional approach, researchers include these groups in a regression analysis in the form of dummy variables (nine mobility groups produce eight dummy variables and estimates). Conclusions on mobility effects are then reached by comparing the effects of the dummy variables representing the mobile groups to an immobile reference category. These mobile categories differ, however, in terms of *both* their experienced mobility *and * their social position of origin and/or destination. Consequently, the estimated mobility effects conflate the effects of social mobility and those of the positions of origin and destination. It, therefore, remains unclear whether the identified effects are indeed due to mobility. Indeed, using this method, it is possible that significant effects are identified, even when no true mobility effects are present.

A similar approach differentiates between upwardly mobile, downwardly mobile and immobile individuals, while controlling for the social position of origin (e.g. Campos-Matos and Kawachi 2015). As Campos-Matos and Kawachi correctly argue, ‘Controlling for parent’s educational achievement yields mobility coefficients that can be interpreted as independent from social group of origin’ (2015 p 242). This approach *does not,* however, yield mobility coefficients that are independent from the social position of *destination*. It, therefore, results in mobility effects that are conflated with destination effects. Again, it is unclear whether the effects identified in such an analysis truly represent mobility effects. This problem cannot be solved using a regular regression approach, as it is not possible to model the effects of a measure of social mobility while simultaneously controlling for the positions of destination and origin in such an approach. This is because of the linear dependency of social mobility on both the social position of origin and social position of destination (Blalock 1966).

In contrast to these conventional approaches employed in extant public health research on social mobility effects, DRMs are able to estimate the effects of: (1) mobility *and* (2) position of origin and (3) position of destination. They do this in a parsimonious and easily interpretable manner. DRMs estimate mobility effects as follows:

$$ Y_{ijk} = w \times \mu_{ii} + \left( {1 - w} \right) \times \mu_{jj} + \mathop \sum \nolimits \beta x_{ijkl} + e_{ijk} $$

where *Y*_*ijk*_ is the value of the dependent variable in cell *ij* of the mobility table which has *k* observations. The part of the equation in front of the summation sign specifies the influence of the position of origin (*i*) and destination (*j*). *μ*_*ii*_ is the estimated mean of *Y* in the diagonal cell in the row denoting the position of origin, whereas *μ*_*jj*_ represents the estimated mean for the diagonal cell in the column denoting the position of destination. When, for instance, we estimate *Y* for those who fell from high to low in Table 1 (bottom-left cell), *μ*_*ii*_ refers to the estimate of *Y* in the cell at the bottom right, which is used for the origin effect. Meanwhile, *μ*_*jj*_ refers to the estimate of *Y* in the cell on the top left, which is used for the destination effect.

**Fig. 1:**
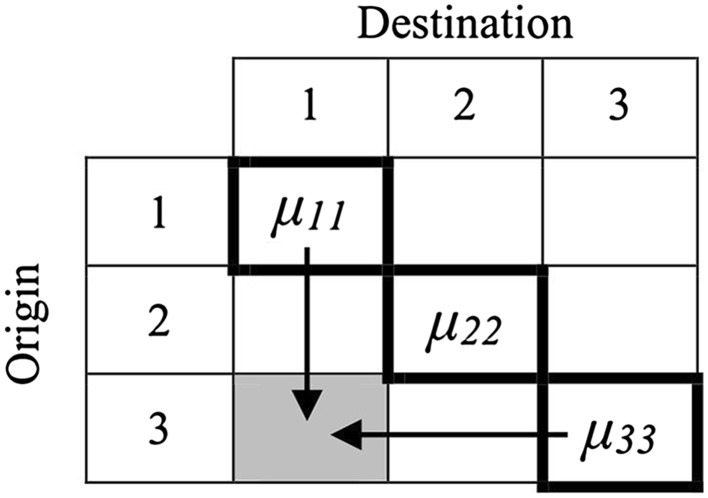
Illustration of the origin and destination effects and the associated w-parameter [effect of origin (*μ*_33_) and destination (*μ*_11_) for the shaded cell: *w* × *μ*_33_ + (1 − *w*) × *μ*_11_]

The *w*-parameter estimates the strength of the effect of position of origin relative to that of the position of destination, and lies in the interval [0; 1]. The diagonal intercepts, combined with the *w*-parameter, allow us to specify a cell-specific intercept for each off-diagonal cell in the mobility table. For example, if the *w*-parameter equals 1 (i.e. the position of destination has no effect, while the position of origin does), the first part of the equation is the same for all the cells with the same position of origin in the mobility table. In contrast, a *w*-parameter that equals 0.5 creates an intercept for each off-diagonal cell that lies between the diagonal intercepts in the column (position of destination) and the row (position of origin) in which this off-diagonal cell is located. This is illustrated in Fig. 1. The estimated intercept for the shaded cell is based on *μ*_11_ and *μ*_33_, as these are the two social positions that individuals in the shaded cell were socialized by. The horizontal arrow illustrates the effect of the social position of origin, while the vertical arrow does the same for the position of destination. Using the *w*-parameter, the two effects are combined in an intercept for this cell. A *w*-parameter of 0.7, for example, would produce an intercept for this cell of 0.7 × *μ*_33_ + (1 − 0.7) × *μ*_11_.

By combining the origin and destination effects in cell-specific intercepts, the model allows us to specify the effect of social mobility *in addition to* the effects of origin and destination (Sobel 1985). This specification is done in the right-hand side of the equation, where covariates are included (represented by the different *x*_*ijk*_ variables and the associated *β*-parameters) which should be interpreted in the same way as in regular regression models. In our models, we include common control variables (age, marital status and native/non-native) and the effect of downwards or upwards mobility as covariates.

Set out below, we contrast the estimates obtained from conventional regression models to those from DRMs, which were estimated using the *Dref* subcommand of the *gnm* package in *R* (Turner and Firth 2007). As our dependent variable is dichotomous, we estimate logistic versions of all the approaches.

## Results

### Modelling social mobility effects on OWOB using conventional logistic regression analyses

Tables 2 and 3 list the odds ratios for both types of conventional logistic regression analysis. The results presented in Table 2 follow on from using dummy variables that indicate mobility groups, while those in Table 3 are obtained by distinguishing upwardly mobile, downwardly mobile and immobile individuals while controlling for the social position of origin.

**Table 2:** Logistic regression models predicting mobility effects on overweight/obesity (body mass index ≥ 25 kg/m^2^), using mobility groups, The Netherlands 2009

Mobility groups	Males			Females		
	OR	95% CI	*p*	OR	95% CI	*p*
Intercept	0.47	[0.31; 0.72]	<0.001	0.35	[0.23; 0.54]	<0.001
Immobile individuals						
High	(Ref.)			(Ref.)		
Medium	1.72	[1.13; 2.63]	0.012	1.75	[1.18; 2.58]	0.005
Low	1.67	[1.11; 2.52]	0.014	2.99	[2.00; 4.48]	<0.001
Downwardly mobile individuals						
High–medium	1.70	[1.02; 2.83]	0.040	1.48	[0.88; 2.49]	0.141
Medium–low	2.40	[1.32; 4.36]	0.004	2.53	[1.41; 4.55]	0.002
High–low	1.61	[0.56; 4.64]	0.379	1.96	[0.71; 5.38]	0.192
Upwardly mobile individuals						
Medium–high	1.07	[0.66; 1.71]	0.794	0.95	[0.60; 1.51]	0.825
Low–medium	1.78	[1.19; 2.65]	0.005	2.01	[1.37; 2.95]	<0.001
Low–high	1.25	[0.77; 2.00]	0.365	1.58	[0.97; 2.57]	0.068
Age (centred)	1.05	[1.03; 1.06]	<0.001	1.04	[1.03; 1.06]	<0.001
Native	0.69	[0.55; 0.87]	0.002	0.70	[0.56; 0.88]	0.002
Marital status						
No partner (ref)						
Married cohabitation	2.37	[1.78; 3.15]	<0.001	1.60	[1.21; 2.10]	<0.001
Unmarried cohabitation	1.41	[1.00; 2.00]	0.052	1.42	[1.00: 2.04]	0.052
No cohabitation	1.35	[0.88; 2.08]	0.169	0.81	[0.50; 1.31]	0.392

Using the International Standard Classification of Education 2011, educational level is classified into three categories: (i) low (categories 1–2), (ii) medium (categories 3–4), and (iii) high (categories 6–8)

**Table 3:** Logistic regression models predicting mobility effects on overweight/obesity (body mass index ≥ 25 kg/m^2^), differentiating upwardly mobile, downwardly mobile and immobile individuals while controlling for social position of origin, The Netherlands 2009

	Males			Females		
	OR	95% CI	*p*	OR	95% CI	*p*
Intercept	0.47	[0.32; 0.70]	<0.001	0.35	[0.24; 0.53]	<0.001
Social mobility						
Immobile	(Ref.)			(Ref.)		
Downwards	1.63	[1.13; 2.35]	0.010	1.52	[1.06; 2.18]	0.024
Upwards	0.84	[0.66; 1.07]	0.161	0.60	[0.48; 0.76]	<0.001
Social position of origin						
Low	1.79	[1.25; 2.58]	0.002	3.07	[2.14; 4.39]	<0.001
Medium	1.50	[1.07; 2.09]	0.017	1.68	[1.21; 2.31]	0.002
High	(Ref.)			(Ref.)		
Age (centred)	1.05	[1.03; 1.06]	<0.001	1.04	[1.02; 1.06]	<0.001
Native	0.70	[0.55; 0.88]	0.002	0.71	[0.57; 0.88]	0.002
Marital status						
No partner (ref)						
Married cohabitation	2.36	[1.78; 3.13]	<0.001	1.61	[1.22; 2.12]	<0.001
Unmarried cohabitation	1.41	[1.00; 2.00]	0.052	1.42	[0.99; 2.03]	0.054
No cohabitation	1.35	[0.88; 2.07]	0.172	0.81	[0.50; 1.30]	0.381

Using the International Standard Classification of Education 2011, educational level is classified into three categories: (i) low (categories 1–2), (ii) medium (categories 3–4), and (iii) high (categories 6–8)

In Table 2, we follow previous studies (Boylan et al. 2014; Chaparro and Koupil 2014; Gigante et al. 2008; Kuntz and Lampert 2012) in taking the ‘always advantaged group’ as the reference category: these are the immobile respondents in the highest educational category. The analysis of the male respondents first shows the well-established social gradient in OWOB: the immobile in the middle (OR = 1.72; *p* = 0.012) and low (OR = 1.67; *p* = 0.014) positions have greater odds of being overweight or obese than the reference category. Second, two mobility effects are found for downwardly mobile males; that is, for those who moved from high to medium (*p* = 0.040) and those who moved from medium to low (*p* = 0.004), but not for those who moved from high to low (*p* = 0.379). Third, a comparison of the upwardly mobile to the reference category indicates a mobility effect for those who moved from low to medium (*p* = 0.005), but not for those who moved from medium to high (*p* = 0.794) and from low to high (*p* = 0.365).

The analysis of the female respondents also demonstrates the well-documented social gradient in OWOB, given the odds ratios for the immobile subjects in the lowest (2.99; *p* < 0.001) and medium (1.75; *p* = 0.005) positions compared to those in the highest position. The analysis indicates a mobility effect (*p* = 0.002) for downwardly mobile females who move from medium to low. In addition, there is a mobility effect for upwardly mobile females who move from low to medium (*p* < 0.001).

Overall, the logistic regression analyses using the mobility groups presented in Table 2 indicate that social mobility effects on OWOB exist for both males and females.

Table 3 presents the results of a second conventional approach, namely logistic regression analyses which distinguish upwardly mobile, downwardly mobile and immobile individuals while controlling for the social position of origin. This analysis also identifies substantial mobility effects. For the male respondents, it suggests that there is a downwards mobility effect on OWOB (*p* = 0.010), while for the females, both upwards and downwards mobility have an impact. According to this analysis, upwardly (*p* < 0.001) and downwardly (*p* = 0.024) mobile females differ significantly in terms of their odds of being OWOB compared to immobile women.

### Modelling social mobility effects on OWOB using Diagonal Reference Models

Table 4 sets out the coefficients for the DRMs. In the first model, we include the effect of downwards mobility (see the upper part of the table), while Model 2 includes the effect of upwards mobility (see the lower part of the table).

**Table 4:** Logistic Diagonal Reference Models predicting mobility effects on overweight/obesity (body mass index ≥ 25 kg/m^2^), The Netherlands 2009

	Males			Females		
	Coef	95% CI	*p*	Coef	95% CI	*p*
*Model 1*						
Diagonal intercepts^a^						
*µ*_11_: low	0.73	[0.55; 0.97]	0.030	0.98	[0.72; 1.34]	0.901
*µ*_22_: medium	0.74	[0.52; 1.03]	0.070	0.55	[0.38; 0.78]	<0.001
*µ*_33_: high	0.41	[0.28; 0.60]	<0.001	0.31	[0.21; 0.47]	<0.001
*w*: weight of origin^b^	0.35	[−0.12; 0.82]		0.32	[0.01; 0.64]	
*β*: covariates^c^						
Downwards mobility	1.27	[0.86; 1.88]	0.227	1.03	[0.66; 1.60]	0.900
Age (centred)	1.05	[1.03; 1.06]	<0.001	1.04	[1.02; 1.06]	<0.001
Native	0.68	[0.54; 0.86]	0.001	0.70	[0.55; 0.89]	0.004
Marital status						
No partner (ref)						
Married cohabitation	2.38	[1.80; 3.15]	<0.001	1.59	[1.19; 2.12]	0.002
Unmarried cohabitation	1.42	[1.01; 2.00]	0.047	1.41	[0.97; 2.05]	0.073
No cohabitation	1.36	[0.90; 2.06]	0.147	0.81	[0.48; 1.34]	0.419
*Model 2*						
Diagonal intercepts^a^						
*µ*_11_: low	0.75	[0.56; 1.00]	0.048	1.01	[0.73; 1.38]	0.962
*µ*_22_: medium	0.73	[0.51; 1.08]	0.093	0.57	[0.40; 0.82]	0.003
*µ*_33_: high	0.43	[0.30; 0.63]	<0.001	0.32	[0.22; 0.48]	<0.001
*w*: weight of origin^b^	0.11	[−0.56; 0.77]		0.43	[−0.02; 0.89]	
*β*: covariates^c^						
Upwards mobility	1.05	[0.78; 1.40]	0.792	0.87	[0.58; 1.32]	0.531
Age (centred)	1.05	[1.03; 1.06]	<0.001	1.04	[1.02; 1.06]	<0.001
Native	0.70	[0.56; 0.88]	0.002	0.70	[0.55; 0.89]	0.004
Marital status						
No partner (ref)						
Married cohabitation	2.37	[1.79; 3.15]	<0.001	1.59	[1.19; 2.13]	0.002
Unmarried cohabitation	1.41	[1.00; 1.99]	0.049	1.41	[0.97; 2.06]	0.071
No cohabitation	1.37	[0.90; 2.07]	0.138	0.82	[0.49; 1.35]	0.436

Using the International Standard Classification of Education 2011, educational level is classified into three categories: (i) low (categories 1–2), (ii) medium (categories 3–4), and (iii) high (categories 6–8)

^a^The diagonal intercepts are odds

^b^The *w*-parameter is a weight parameter. No valid *p* value can be computed, as the weight parameter is constrained between zero and one

^c^The coefficients for the covariates are odds ratio’s

The males in Model 1 are considered first. The diagonal intercepts represent the estimated means for immobile individuals in the three discerned positions (i.e., *µ*_11_, *µ*_22_, *µ*_33_). So, 0.73, for example, represents the odds of being OWOB for immobile males with the lowest educational level. Meanwhile, the immobile men who are in the highest position in the social hierarchy have lower odds of being OWOB (0.41). These diagonal intercepts are used to estimate the origin and destination effects for mobile males.

The *w*-parameter indicates to what extent mobile men are influenced by origin effects relative to destination effects. The results show that the influence of origin is not significantly greater than that of destination, as the *w*-parameter of 0.35 does not significantly differ from 0.5. This means that we cannot reject the null hypothesis (*w* = 0.5), which states that mobile males are influenced to the same extent by their positions of origin and destination. As an example, the origin and destination effects for individuals who move from high to low are represented in the odds for OWOB that lie between the diagonal intercepts of low and high (=0.35 × 0.41 + (1 − 0.35) × 0.73). These influences of origin and destination apply to all the mobile males. The model also tests if downwards social mobility has an additional effect on the odds of being OWOB, but this effect is not identified (*p* = 0.227).

The same conclusions are reached for the downwards mobility of females (Model 1) and the upwards mobility of males and females (Model 2). First, the diagonal intercepts differ from one another in both models, indicating a social gradient with respect to OWOB. Second, as the *w*-parameters do not significantly differ from 0.5, we cannot reject the null hypothesis (*w* = 0.5), which states that mobile individuals are influenced to the same extent by their positions of origin and destination. Third, when origin and destination effects are accounted for by applying DRMs, no impact of either downwards mobility (among females) or upwards social mobility (among males and females) on OWOB is found (*p* = 0.900, *p* = 0.792 and *p* = 0.531).

## Discussion

Various studies on the health consequences of SEP focus on social mobility effects, in addition to the health consequences of the social positions of origin and destination. The current study demonstrates that the methods conventionally used in public health research to estimate these mobility effects may produce invalid results. This is likely to occur because the estimated mobility effects in conventional approaches conflate these effects with effects of the positions of origin and destination. In contrast, DRMs calculate these mobility effects simultaneously with origin and destination effects, preventing such conflation.

To illustrate our argument, we focused on a specific health outcome: OWOB. We first estimated intergenerational educational mobility effects on OWOB in the Netherlands using conventional logistic regression analyses. We then compared the results to those produced by DRMs. The former suggested that mobility effects exist, reminiscent of the findings of 13 of the 15 studies of mobility effects on BMI or OWOB outlined in the Introduction. In contrast, the analyses with DRMs found no social mobility effects in our sample.

This discrepancy in the results sheds doubt on the validity of the findings of studies that estimate mobility effects using conventional regression analyses. Given that the method is the only difference between our conventional logistic regression analyses and those using DRMs, the discrepancy in the results in our study can only be caused by the conflation of mobility effects with origin and destination effects in the former type of analysis. It remains an open empirical question whether the previously reported health effects of social mobility also result from such conflation. Using DRMs is necessary to answer this question, not only when it comes to studies on the link between intergenerational educational mobility and BMI or OWOB, but for research on the effects of all types of social mobility on all health behaviours and outcomes.

A similar argument can be made for studies directed at other kinds of ‘structural mismatch’ than social mobility, such as inconsistencies in educational attainment or occupational status among parents or between partners (cf. Eeckhaut et al. 2013; Willekens et al. 2014), or between an acquired level of education and actual income (Lenski 1954). Some individuals have, for instance, a substantially lower or higher income than the average return for their level of education (Peter et al. 2016). DRMs are needed to validly estimate the effects of this inconsistency, as they allow estimations to be simultaneously made of the effects of education and income *and* the inconsistency between them. Conventional methods that model status inconsistency using categories created by specific combinations of education and income (e.g. Peter et al. 2016) may produce invalid results.

We aimed to demonstrate the advantage of DRMs over conventional methods for estimating mobility effects in public health. We provided an empirical analysis to illustrate this methodological argument. Various limitations need to be taken into account. We used a sample with a restricted age range (18–45), focused on merely one health outcome (OWOB) and one kind of social mobility (intergenerational educational mobility), in just one country (the Netherlands). In addition, we did not model possible mediators that link intergenerational educational mobility to OWOB, and we did not test whether social mobility effects are affected by the time people have spent in their position of origin (cf. Houle 2011). The modest scope of our study enabled us to focus on our methodological message. Future substantive research on social mobility and health could improve upon these limitations.

Applying DRMs in future research on the health consequences of social mobility or other types of structural mismatch is crucial for at least two reasons. First, it can uncover whether the conventional approaches for estimating the health effects of social mobility and other structural mismatches yielded false positives in extant studies. Second, it may well be that various types of structural mismatch prove to affect various health behaviours and outcomes if DRMs are used, including the ones addressed in the studies discussed in our Introduction and the Methods section. Validly estimating the health effects of social mobility and other structural mismatches is an important contribution to providing effective policy responses to contemporary social gradients in health.

Overall, we strongly recommend that future studies of the health consequences of social mobility and other structural mismatches in public health use DRMs.
